# A primer on heart rate variability: use in neuroscience and psychiatry

**DOI:** 10.1038/s44277-026-00072-1

**Published:** 2026-10-15

**Authors:** Courtney C. Louis, Antonia V. Seligowski

**Affiliations:** 1 Department of Psychiatry, Harvard Medical School, Boston, MA USA; 2 Department of Psychiatry, Massachusetts General Hospital, Boston, MA USA

**Keywords:** Risk factors, Cardiovascular biology

Heart rate variability (HRV) is widely recognized as a key mechanism that contributes to several health outcomes. We provide a broad overview of HRV, its clinical and translational relevance, and important factors to consider when measuring HRV in research endeavors.

## What is heart rate variability?

HRV is the variation of time intervals between successive heartbeats. A complex interplay of physiological processes influences variability between successive heartbeats, including neural, respiratory, and autonomic dynamics [1]. A fundamental goal of a living organism is to constantly adapt to a changing environment, and HRV is generally considered to be indicative of the ability to flexibly adapt to internal and external demands. More variability reflects greater control of the vagus nerve over the heart’s sinoatrial node (the heart’s endogenous pacemaker) [2]. The majority of vagus nerve fibers are afferent and send signals to the nucleus tractus solitarius (NTS), locus coeruleus (LC), and prefrontal cortex (PFC). These neural regions also send reciprocal signals to the vagus nerve, thereby regulating cardiac output. As such, HRV is considered a measure of “top-down” regulatory abilities.

## How is HRV measured?

HRV is quantified by measuring inter-beat intervals, typically assessed using non-invasive methods such as electrocardiography (ECG) or photoplethysmography (PPG). ECG is considered the gold standard because it provides a more precise measure of the heart’s electrical activity. PPG is typically used in wearable devices and measures light absorption by shining green, red, near-infrared light into the skin to assess variations in blood circulation. Each method carries advantages and disadvantages, and the use of either may depend on study goals and resources. While ECG is more precise, it can be more costly, and clinical ECG may require in-person assessments in order to transmit signals to a computer. PPG offers advantages in accessibility and cost, but it is more susceptible to artifacts, including motion, sweat, and skin tone. Nonetheless, there is encouraging data on concordance of certain HRV metrics between PPG and ECG devices, particularly during sleep [3].

The most common HRV metrics are computed in the *time* or *frequency* domain [2]. The time-domain uses filtered RR intervals (the time between successive R peaks of the ECG), known as NN intervals (normal-to-normal, such that abnormal beats are filtered out). Common time-domain HRV metrics include deviation-based indices, such as the standard deviation of NN intervals (SDNN) and difference-based indices, such as the root mean square of successive differences (RMSSD). On the other hand, frequency-domain metrics use power spectral analysis to decompose the variance in heart rhythm oscillations into different frequency bands. Common frequency-domain HRV measures include high-frequency HRV (often referred to as respiratory sinus arrhythmia), low-frequency HRV (often linked to baroreceptor activity), and the low/high frequency ratio. Non-linear HRV methods may also be of interest, such as entropy analyses that examine the pattern of HRV amplitudes (where higher entropy reflects higher signal irregularity), and machine learning can be leveraged to automate analysis of large, longitudinal HRV datasets that are common in wearables research [4]. Use of a particular metric may be guided by the autonomic characteristic of interest, as each may reflect different aspects of parasympathetic and sympathetic nervous system activity and their relative balance [3].

## How is HRV altered in psychiatric diseases?

Decades of evidence have demonstrated that individuals with various forms of psychopathology demonstrate altered HRV, both at rest and in response to stress. In general, resting HRV tends to be lower among those with depression, anxiety, posttraumatic stress disorder, and psychotic conditions [5, 6]. During stress, healthy controls experience temporary decreases in HRV that are followed by increases (i.e., the regulatory system adjusts to the stress), while people with psychiatric disorders appear to have more significant decreases and less rebounding. As low HRV is implicated in cardiometabolic and other physical health conditions [5], it is an important physiological disease mechanism in people with psychiatric disorders. The clinical significance of HRV in psychopathology is an active area of research, with varying perspectives on whether HRV should be conceptualized as a biomarker of psychopathology (e.g., anxiety), a trait-like correlate of autonomic regulatory function, or a mechanistic treatment target (e.g., biofeedback). However, most theoretical frameworks converge on the view that reduced HRV is an emergent property of disrupted brain-heart interactions, reflecting autonomic strain and diminished regulatory capacity [7].

In addition, including HRV measurement in clinical research has strong translational relevance. To model psychiatric stress, many animal studies induce stress or threat through fear conditioning and chronic social defeat stress paradigms. Overall, results are consistent with the human literature, such that rodents exposed to stress demonstrate decreased HRV compared to controls [8, 9]. Given the highly conserved neural circuitry of stress-responsive brain regions that control HRV (e.g., prefrontal cortex, amygdala), animal models can and should be leveraged when considering HRV in humans.

## Biological and social determinants of HRV

Many of the psychiatric conditions that are linked to reduced HRV also disproportionately burden those assigned female sex at birth. For example, female participants exhibit higher HRV and stronger associations between HRV and emotion regulation difficulties compared to male participants [10]. A recent meta-analysis found reduced vagal control during menstrual phases with higher ovarian hormone concentrations compared to phases with lower concentrations. There is also a significant decline in vagal control during vasomotor symptom episodes in perimenopause. Furthermore, recent research has highlighted racial/ethnic differences in HRV. Compared with White individuals, Black individuals tend to evidence higher HRV and higher peripheral resistance. This has been referred to as the “cardiovascular conundrum” and may reflect a psychophysiological compensatory response to increased stress exposure [11]. Together, these patterns suggest that it is critical to account for biological and social determinants when studying the functional significance of HRV in psychopathology and stress.

## Considerations for research

**Fig. 1:**
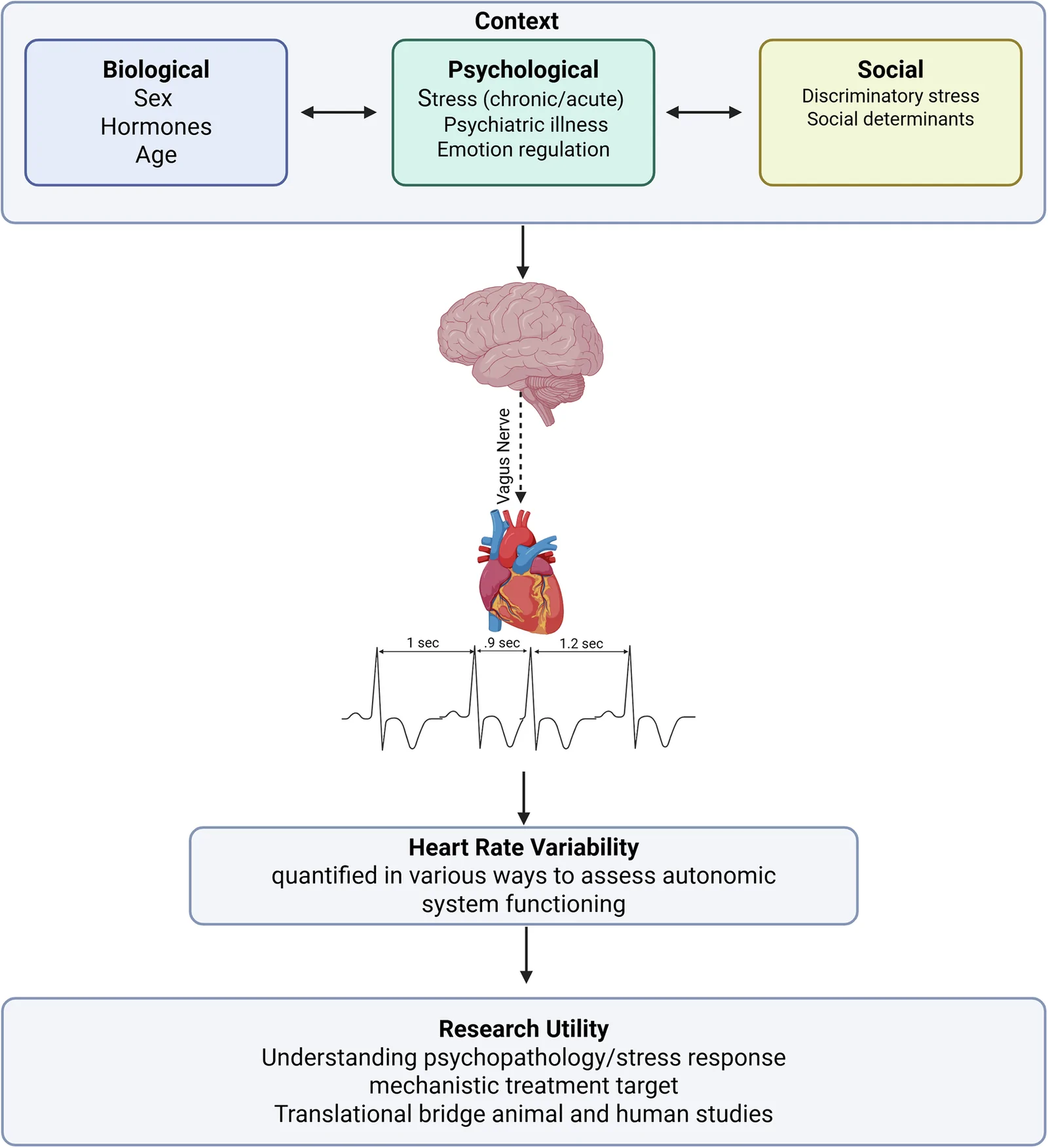
Conceptual figure demonstrating that biological, psychological, and social determinants influence heart rate variability (HRV). Biological (e.g., sex, gonadal hormones, age), psychological (e.g., stress, psychiatric illness, emotion regulation), and social (e.g., discriminatory stress, social determinants) influences brain-autonomic interactions. HRV, calculated with beat-to-beat interval variations, can be quantified in various ways (e.g., time-domain, frequency-domain). HRV has broad research utility, including as a tool for understanding psychopathology, as a mechanistic treatment target, and as a translational bridge between animal and human studies. Created in https://BioRender.com.

HRV is a widely recognized marker of autonomic function that contributes to psychopathology and cardiometabolic outcomes. There are many ways to assess and compute HRV, and the appropriate metric depends on the research question and the aspect of autonomic function (e.g., sympathetic and/or parasympathetic) of interest. We encourage consideration of sex as a biological variable, along with recognition that racial/ethnic differences (particularly for PPG sensors) and socioeconomic factors may affect measurement and contribute to variability in HRV findings (see Fig. 1). Researchers interested in HRV may consider it as a dynamic reflection of regulatory capacity that may change as a function of biological, psychological, and social contexts.
